# Association between respiratory disease pathogens in calves near feedlot arrival with treatment for bovine respiratory disease and subsequent antimicrobial resistance status

**DOI:** 10.3389/fvets.2024.1416436

**Published:** 2024-07-23

**Authors:** Jennifer N. Abi Younes, John R. Campbell, Sheryl P. Gow, Amelia R. Woolums, Cheryl L. Waldner

**Affiliations:** ^1^Department of Large Animal Clinical Sciences, Western College of Veterinary Medicine, University of Saskatchewan, Saskatoon, SK, Canada; ^2^Canadian Integrated Program for Antimicrobial Resistance Surveillance, Public Health Agency of Canada, Saskatoon, SK, Canada; ^3^Department of Pathobiology and Population Medicine, College of Veterinary Medicine, Mississippi State University, Starkville, MS, United States

**Keywords:** bovine respiratory disease, bacterial culture, antimicrobial susceptibility, antimicrobial use, sampling, antimicrobial treatment

## Abstract

**Introduction:**

This study assessed the risk of first treatment for bovine respiratory disease (BRD) given detection of nasopharyngeal bacteria (*Mannheimia haemolytica*, *Pasteurella multocida,* and *Histophilus somni*) and corresponding likelihood of antimicrobial susceptibility (C/S) at two time points during the early feeding period. Relationships between C/S results and later treatment for BRD were evaluated at both the calf-level and pen-level. The association between calf-level and pen-level C/S findings during the early feeding period and subsequent C/S results at BRD treatment were also reported.

**Methods:**

Auction-sourced, recently-weaned beef calves (*n* = 1,599 steers) were placed in adjacent feedlot pens (8 × 100 calves) in two subsequent years. Deep nasopharyngeal (DNP) swabs were collected from all calves at time of arrival processing (1DOF) and before metaphylaxis administration with either tulathromycin or oxytetracycline, 12 days later (13DOF), and at the time of first treatment for BRD. All samples were tested for C/S.

**Results:**

Several pen-level and individual calf-level C/S measures of interest were associated with future treatment for BRD and C/S at the time of treatment. The median DOF for first BRD treatment was 24 days following tulathromycin metaphylaxis and 11 days following oxytetracycline. Overall, sampling at 13DOF resulted in the best fit for more models of subsequent treatment for BRD and C/S results at BRD treatment than for sampling at arrival. In individual calves, recovery of *M. haemolytica*, *P. multocida*, or *H. somni* at 13DOF was associated with subsequent treatment for BRD within 45DOF. Pen-level prevalence of *Pasteurellacea* bacteria with tetracycline or macrolide resistance at arrival and 13DOF were associated with detection of bacteria with antimicrobial resistance (AMR) at BRD treatment, as were individual calf results at 13DOF.

**Discussion:**

These findings suggest that the bacteria and AMR outcomes recovered from cattle near two weeks on feed can inform the prediction of future BRD risk and concurrent antimicrobial susceptibility results at time of first BRD treatment. Notably, the associations between pen-level C/S results from previous testing and corresponding findings in calves with BRD from the same pen suggested potential testing strategies to inform antimicrobial use protocols for feedlot cattle.

## 1 Introduction

The incidence of bovine respiratory disease (BRD) in North American feedlots has remained relatively consistent over the last 45 years despite extensive research into the disease process and intervention strategies (1). The multifactorial nature of BRD complicates progress in prevention, control, and treatment (2). In addition to several viruses and *Mycoplasma bovis*, the bacteria *Mannheimia haemolytica*, *Pasteurella multocida*, and *Histophilus somni* have consistently been implicated in the etiology of BRD.

Antimicrobials continue to be more effective than vaccines, nutraceuticals, or intranasal bacterial therapeutics for the control of BRD (3, 4). However, calves at risk of BRD are empirically treated with antimicrobials based on historical clinical data, limited animal history, experience, and available therapeutic data. The limited use of laboratory data is due in part to the turnaround time to receive results from culture and antimicrobial susceptibility (C/S) tests from clinical cases, which can be several days. Due to the inability to obtain results in a timely fashion, laboratory diagnostics are generally reserved for select BRD mortalities or outbreaks of clinical cases that are refractory to treatment. Several studies on BRD mortalities in conventional feedlots in Alberta and the United States have identified multi-drug-resistant *Pasteurellaceae* strains (5–7). However, bacteria from cattle sampled at feedlot arrival in Western Canada are predominately susceptible to routinely tested antimicrobials (8–10). This suggests antimicrobial resistance (AMR) observed in bacteria from samples from deceased or repeatedly treated cattle might not be representative of bacteria in calves treated for the first time for BRD during the early feeding period. With growing concern about antimicrobial resistance (AMR) in both humans and animals (8), the need for evidenced-based antimicrobial treatment protocols is increasing.

Studies utilizing laboratory diagnostics show bacteria recovered from the respiratory tract differ between healthy and BRD-affected cattle (9–12). Results regarding the predictability of respiratory bacteria recovered at feedlot arrival and associations with later BRD diagnosis (13–15) and subsequent clinical resolution (16) have varied. To the authors’ knowledge, the utility of using a sampling time shortly after feedlot arrival to predict future disease risk or culture patterns at the time of BRD treatment had not been reported at the time of this publication. By allowing time for the respiratory microbial community to adapt following feedlot placement, comingling and metaphylaxis (17), C/S data from a slightly later sampling time might better indicate the subsequent likelihood of BRD treatment and more aptly inform antimicrobial use (AMU) for BRD than data collected at arrival.

Additionally, while the recovery of respiratory bacteria from individual feedlot calves has been explored as a predictor of BRD (13, 15), there have been no prior reports of utilizing pen-level prevalence of respiratory bacteria to examine potential associations with future BRD or AMR outcomes (7). As feedlot cattle are managed as groups, the prevalence of bacteria and AMR within the pen cohort has the potential to influence an individual calf’s risk of acquiring BRD-associated bacteria, including those that are resistant to antimicrobials. Indeed, findings from Shane et al. (18) favor the hypothesis of clinical BRD transmission between pen mates, suggesting the communicability of BRD pathogens between diseased and healthy cattle. Additionally, studies using pulsed field gel electrophoresis (PFGE) and whole genome sequencing (WGS) have illustrated horizontal transmission of BRD bacteria and AMR genes between cattle (19, 20), and further support the association between a calf’s health status and that of its pen mates.

The first objective of this study was to assess whether the recovery of bacterial pathogens (*M. haemolytica*, *P. multocida*, and *H. somni*) and antimicrobial susceptibility results in feedlot calves at arrival processing and 13 days on feed (1DOF and 13DOF) could predict future risk of first treatment for BRD. The second objective was to describe the association between C/S results from the early feeding period (1DOF and 13DOF) and C/S results at subsequent first treatment for BRD. For each question, C/S results from individual calves were compared to pen prevalence to identify the sampling strategy with the potential to best inform antimicrobial protocols for BRD treatment.

## 2 Materials and methods

### 2.1 Ethical statement

The research protocols and procedures for this study were approved by the University of Saskatchewan Animal Care Committee (AUP 20190069).

### 2.2 Study population

The calves treated for BRD and evaluated in this study were also part of a study describing changes in the prevalence of *M. haemolytica*, *P. multocida*, and *H. somni* and antimicrobial susceptibility results in feedlot calves at two time points during the early feeding period (21). Further detail on the study population, including comprehensive C/S data for samples collected for all calves from the present study at 1DOF and 13DOF are presented elsewhere (21) with only the information necessary for the objectives of this paper summarized in this report.

Briefly, 1,600 mixed-origin, auction-derived steers were enrolled. One calf was down at the time of processing and remained in the hospital pen for the duration of the study, resulting in 1,599 available calves. One hundred animals were sourced weekly for eight weeks from the end of September to mid-November of 2020 and again in 2021 (800 calves per year). Animals were maintained in pens with their arrival cohort of 100 calves to 45DOF. At 1DOF, mean calf weight in 2020 was 253 kg (556 lbs) [range: 211–291 kg (464–640 lbs), standard deviation: 14 kg (31 lbs)]. Lighter calves were targeted in 2021 with a mean calf arrival weight of 225 kg (496 lbs) [range: 160–315 kg (351–694 lbs); standard deviation: 15 kg (34 lbs)]. Calves were housed in eight outdoor, dirt floor pens with a maximum holding capacity of 100 calves/pen and designed as per the Canadian guidelines for feedlot cattle (22). The first four pens and the last four pens were connected by consecutive cross-fences, while a building separated the initial four pens from the final four. Adjacent pairs of pens shared fence line watering bowls. Calves were fed a diet meeting the National Research Council (NRC) suggested requirements for beef cattle (21, 23).

Calves were processed using protocols typical for moderate- to high-risk calves in commercial feedlots. All calf cohorts from 2020 (*n* = 800; Pens 1–8) received injec tulathromycin (Draxxin^®^, Zoetis Inc., Florham Park, NJ; 2.5 mg/kg) as metaphylaxis on arrival. Half of the 2021 cohort (*n* = 400; Pens 9–11, 16) received injectable oxytetracycline (Oxyvet^®^200 LA, Vetoquinol, Lavaltrie, QC; 20 mg/kg) on arrival and the other half (*n* = 400; Pens 12–15) received tulathromycin.

Three deep nasopharyngeal (DNP) swabs were collected from alternating nostrils from each calf. The three DNP swabs were pooled into a designated 15 mL vial containing 3 mL of liquid Amies transport medium. All calves were sampled at two time points: at arrival processing (1DOF) prior to metaphylaxis administration and again 12 days later (13DOF). A subset of calves from each pen cohort were also sampled at a third time point near 36DOF (10 calves/pen in 2020 and 30 calves/pen in 2021). The only exception to this approach was in pen 16 where a rise in BRD morbidities and mortalities associated with *H. somni* resulted in 20 calves being sampled at 30DOF in 2021 prior to mass medication with oxytetracycline [(Vetoquinol, Oxyvet^®^ 200 LA, Lavaltrie, QC) subcutaneously at a dose of 20 mg/kg body weight]. The 20 calves sampled prior to treatment were used for analysis of results near 36DOF (as opposed to the scheduled third sampling time at 36DOF, which occurred after mass treatment).

### 2.3 Treatment of calves with BRD

Experienced feedlot personnel monitored animals daily for signs of illness. Calves exhibiting signs of BRD were identified using a standardized DART (depression, appetite, respiratory system, temperature) BRD clinical scoring system (Supplementary Table S0) (24). The severity of clinical signs was graded using a numerical scale ranging from 0 (clinically normal) to 4 (moribund). To meet the BRD case definition and receive treatment, calves required a score of 1 or 2 with a rectal temperature ≥ 40°C, or a score of 3 or 4 regardless of temperature (and with no other obvious causes of illness) (25).

Calves that met the case definition for requiring BRD treatment were sampled as described by trained feedlot personnel at time of BRD treatment, prior to antimicrobial administration. DNP swabs from calves with BRD were stored and refrigerated for up to 72 h until transport to the laboratory for immediate processing. An aliquot of the pooled sample from the three DNPs for each calf was submitted for bacterial C/S.

For calves that received tulathromycin metaphylaxis, a post-metaphylaxis interval (PMI) of seven days was observed during which no cattle were eligible for treatment. The BRD treatment regimen for these calves was florfenicol (300 mg/mL) and flunixin meglumine (16.5 mg/mL) (Resflor Gold^®^, Merck Animal Health, Rahway, NJ) subcutaneously administered at 6 mL/45.4 kg body weight. For calves that received oxytetracycline metaphylaxis, a five-day PMI was observed; calves requiring treatment for BRD received tulathromycin (Draxxin^®^, Zoetis Inc., Florham Park, NJ) at a dose of 2.5 mg/kg body weight. Calves were returned to their home pen following treatment.

As previously reported (21), three calves died from BRD in 2020 and one calf died from bloat. In 2021, eight calves died from BRD; five were from pen 16. Other mortalities in 2021 included four cases of bloat and one euthanasia due to neurologic symptoms, which was also found to have lung lesions. A bovine field services veterinarian or anatomic pathologist from the Western College of Veterinary Medicine necropsied all cattle that died.

### 2.4 Microbiology methods

A detailed description of the laboratory procedures has been reported (21). Briefly, all samples collected at days 1 and 13 on feed underwent same-day processing at the University of Saskatchewan. The samples were vortexed for 1 min and a 300 μL aliquot from a pool of the three samples was submitted to Prairie Diagnostic Services, Inc. (Saskatoon, SK, PDS). Culturing for specific bacteria involved 10 μL inoculations on Columbia agar with sheep blood and chocolate agar, incubated at 35°C for 18 h in a 5% CO_2_ environment. Colonies displaying desired morphologies were selected and confirmed by a matrix-assisted laser desorption/ionization time-of-flight mass spectrometry (MALDI-TOF MS) Microflex LT instrument (Bruker Daltonik, Bremen, Germany), and MALDI-TOF MS Biotyper software (Bruker Corporation, Billerica, MA). Each day of sample setup and every new media batch underwent processing with both positive and negative controls, utilizing *Staphylococcus aureus* ATCC 29213, *Escherichia coli* ATCC 25922, and *Histophilus somni* ATCC 700025. Only MALDI-TOF MS identification scores ≥2, ensuring a secure species-level identification, were considered for further laboratory analysis.

Antimicrobial susceptibility testing (AST) was conducted using a commercially available bovine serial broth microdilution panel (Vet Bovine AST BOPO7F Plate, Thermo Fisher Scientific™, Mississauga, ON) on the Sensititre™ platform. Quality control procedures were completed following manufacturer instructions using *E. coli* ATCC 25922, *S. aureus* ATCC 29213, and *H. somni* ATCC 700025. Minimum inhibitory concentration (MIC) plates were placed and read on a BIOMIC^®^ V3 microplate reader (Giles Scientific Inc., Santa Barbara, CA). The MICs for each antimicrobial were compared against breakpoints designated by the Clinical and Laboratory Standards Institute where available (26). Isolates with MIC values considered intermediate were categorized as “susceptible” for all analyses.

### 2.5 Statistical analysis

Data were managed in a commercial spreadsheet program (Microsoft Excel, version 2,401, Microsoft Corporation, Redmond, WA). Analyses were completed using the Stata^®^ statistical software package (Stata/IC, version 16.1, StataCorp LLC, College Station, TX, United States) unless otherwise noted. Data pertaining to detection of *M. haemolytica*, *P. multocida*, and *H. somni* and corresponding antimicrobial susceptibility were summarized at the calf level rather than the isolate level (i.e., number of calves with tulathromycin-resistant *M. haemolytica* as opposed to number of *M. haemolytica* isolates resistant to tulathromycin). Additionally, pen-level prevalence (i.e., number of calves with bacteria detected/total number of calves sampled per pen) were reported for all outcomes of interest.

Cox proportional hazards models were used to estimate the difference among metaphylaxis types and study years in the rate of first BRD treatment within the first 45 days on feed. Clustering by pen was accounted for using a shared frailty term. Differences in median times to first BRD treatment among metaphylaxis types and study years were examined with Kruskal-Wallis tests.

The regression models examined are summarized in Table 1 and detailed as follows. For calf-level regression modeling, outcomes of interest included the likelihood of a calf being treated for BRD and culture and antimicrobial susceptibility test (C/S) results at the time of first treatment for BRD and at 36DOF. Generalized estimating equations (GEE) were chosen as a quasi-likelihood approach for analyzing data with correlated outcomes. The GEE models were fit as population-averaged generalized linear models with a logit link function and binomial distribution. The GEE models accounted for clustering within pens by specifying pen as a panel term with an exchangeable within group structure for the correlation matrix and robust variance. All GEE models included a fixed effect to account for sampling year and injectable metaphylaxis used (i.e., 2020 calves were administered tulathromycin metaphylaxis, whereas half of the 2021 calves were administered tulathromycin metaphylaxis and half were administered oxytetracycline metaphylaxis). If GEE models with the fixed effect for sampling year and injectable metaphylaxis failed to converge and generate effect estimates due to zero counts in one or more year/metaphylaxis groups, an unconditional GEE model without the fixed effect was examined instead. If the unconditional GEE model also failed to converge and generate effect estimates, exact logistic regression models (SAS^®^ version 9.4, Cary, NC) were used to estimate odds ratio, 95% confidence intervals, and exact *p-*values.

**Table 1:** Summary of all BRD and culture and susceptibility (C/S) outcomes and BRD onset times considered when examining associations with C/S results for deep nasopharyngeal (DNP) samples collected at 1DOF and 13DOF for individual animal-level and pen-level analysis.

Outcome of interest		Unit of analysis	
		Individual	Pen
1st BRD treatment	**Timing of BRD treatment:**	**Outcome:**	**Outcome:**
		**Individual calf treatment for BRD (Yes/No)**	**Number calves treated per pen/ Number calves at risk per pen**
		**Risk factor:**	**Risk factor:**
		**Matched to calf DNP results from C/S at:**	**Proportion of pen DNP positive for C/S at:**
	Day 1 to 13	1DOF	1DOF
	Day 1 to 45	1DOF	1DOF
	Day 14 to 45	13DOF	13DOF
Culture and susceptibility results at the time of BRD treatment	**Timing of 1st BRD treatment and BRD sample collection:**	**Outcome:**	**Outcome:**
		**DNP C/S—Calf status at BRD treatment (positive/negative)**	**DNP C/S positive at BRD treatment per pen/BRD calves treated per pen**
		**Risk factor:**	**Risk factor:**
		**Matched to calf DNP results from C/S at:**	**Proportion of pen DNP positive for C/S at:**
	Day 1 to 13	1DOF	1DOF
	Day 1 to 45	1DOF	1DOF
	Day 14 to 45	13DOF	13DOF

Culture and susceptibility (C/S) results considered: • recovery of *M. haemolytica*, *P. multocida*, or *H. somni*, • recovery of any BRD bacteria with any AMR, macrolide resistance, or tetracycline resistance.

For all models, potential risk factors of interest included positive culture for *M. haemolytica*, *P. multocida*, or *H. somni*. Additionally, antimicrobial resistance patterns of interested included either *M. haemolytica*, *P. multocida*, or *H. somni* with AMR to tetracycline, macrolides (tulathromycin, gamithromycin, tildipirosin, or tilmicosin), or any of these three *Pasteurellacea* bacteria with AMR to any antimicrobials tested with available CLSI breakpoints. Table 1 provides a summary of primary outcomes with the timing of BRD treatments and sample DNP collection at treatment considered in the analysis, and then for each unit of analysis (individual and pen) lists the respective outcomes, the C/S results considered as potential risk factors and timing of C/S assessment. An example of how to read Table 1 moving from right to left in the first row is as follows: In the first analysis of the likelihood that any individual calf requires first treatment for BRD within 13DOF, we examined C/S results obtained at 1DOF from that calf as a potential individual calf risk factor.

The first models examined whether an individual calf’s C/S results at 1DOF were associated with whether that calf was treated for BRD on or before 13DOF. The same approach was taken to assess whether C/S results at 1DOF were associated with calf treatment within 45DOF. Similar analyses were also used to evaluate the association between C/S results recovered at 13DOF and calf treatment for BRD between 14DOF and 45DOF.

The second set of models examined whether an individual calf’s C/S results at feedlot arrival processing (1DOF) were associated with that calf’s corresponding C/S results at time of first BRD treatment. Separate models were constructed for calves treated at or before 13DOF, and then for any calves requiring BRD treatment during the 45-day study period. The analyses were repeated to evaluate whether culture results at 13DOF were associated with calf C/S results at the time of treatment for those calves treated after 13DOF. Culture results considered in the models included those listed in Table 1. Other fixed effects in this group of models included the combined variable for year and metaphylaxis and days on feed at time of BRD treatment.

The third set of models examined the associations between C/S results at 1DOF and 13DOF, and the likelihood of calves having corresponding C/S results when sampled near 36DOF as described above (Supplementary Tables S1–S3).

The fourth set of models investigated associations between pen-level prevalence of C/S results and the pen-level risk of BRD using GEEs in SAS (SAS^®^ version 9.4, Cary, NC) with a logit link function, binominal distribution, and repeated term for pen to account for clustering of outcomes within pen with an exchangeable correlation structure. The number of calves treated for BRD from each pen cohort was the outcome of interest for each model (numerator) and the total number of calves in the pen (denominator). The risk factors of interest included the pen-level prevalence (16 pens total) of C/S results of interest described in Table 1. Separate models were used to predict BRD for three risk periods (1 to 13DOF, 1 to 45DOF, 14 to 45DOF), using the pen-level prevalence of C/S results of interest, from samples collected at both 1DOF and 13DOF, as previously described for calf-level risk.

The log odds for continuous risk factors in the pen-level models were rescaled to facilitate interpretation and then back transformed. As a result, the odds ratios represent the multiplicative increases in the odds of the outcome of interest for every 5% increment in prevalence of the risk factor. Rescaling was completed only after testing any identified significant associations including C/S prevalences for linear associations with the log odds of the outcome of interest by testing the significance of a quadratic term in the equation. If the quadratic term was significant and the linear assumption failed, then the quasi-information criteria (QIC) of the more complex model with the quadratic term was compared to that of the simple model without the quadratic term. Lower QICs indicate better model fit. If the QIC for the model with quadratic term was lower, the model with the quadratic term was retained. If the QIC was more than 20% higher, the simple model was retained.

Similarly, GEE models using the pen-level prevalence of C/S results at 1DOF and 13DOF were used to predict the future pen C/S prevalence of calves at time of BRD treatment for these risk periods (Table 1).

The fifth set of models included the same specifications and predictor variables as previously stated (Supplementary Table S1) to investigate the pen-level prevalence of C/S results at 1DOF and 13DOF for predicting the likelihood of subsequent pen-level C/S results recovered from calves sampled near 36DOF (Supplementary Table S4). Results of all analysis of C/S collected at 36DOF were summarized in Supplementary Table S5.

A summary of the odds ratios from the regression models for design variables and potential confounders were provided in Supplementary Tables S6–S11. Where both a design variable and the primary risk factor of interest were significant, the model was re-evaluated including an interaction term. The *p*-values for the interaction terms are also reported in Supplementary Tables S7–S11.

For all significant results, the fit and predictive ability of the model were evaluated by developing receiver operating characteristic (ROC) curves and reporting the Area Under the Curve (AUC) (Supplementary Tables S7–S11).

## 3 Results

### 3.1 BRD-treated calves

Eight percent of calves (130/1,599) were treated at least once for BRD within the 45-day study period. The number of calves treated for BRD varied across pens, with a range of 0 to 7/100 calves for the pens that received tulathromycin metaphylaxis and a range of 13 to 35/100 calves for the pens that received oxytetracycline (Table 2). Oxytetracycline-treated calves in 2021 were treated for BRD at twice the rate of both 2020 (HR = 2.04, *p <* 0.001) and 2021 tulathromycin-treated calves (HR = 2.08, *p <* 0.001). However, the rate of BRD treatment was not different between tulathromycin-treated calves in 2020 and 2021 (*p =* 0.93).

**Table 2:** Number and percent (%) of calves receiving first treatment for BRD by pen and year and injectable metaphylaxis treatment administered at arrival processing (1DOF) (*n* = 1,599 calves, *N* = 16 pens).

Year	Pen	Metaphylaxis	Number calves in pen	Number BRD calves	% BRD calves
2020	1	Tulathromycin	100	1	1%
	2	Tulathromycin	100	3	3%
	3	Tulathromycin	100	7	7%
	4	Tulathromycin	100	7	7%
	5	Tulathromycin	100	5	5%
	6	Tulathromycin	99	3	3%
	7	Tulathromycin	100	2	2%
	8	Tulathromycin	100	0	0%
2021	9	Oxytetracycline	100	16	16%
	10	Oxytetracycline	100	25	25%
	11	Oxytetracycline	100	35	35%
	12	Tulathromycin	100	4	4%
	13	Tulathromycin	100	5	5%
	14	Tulathromycin	100	1	1%
	15	Tulathromycin	100	3	3%
	16	Oxytetracycline	100	13	13%

**Figure 1:**
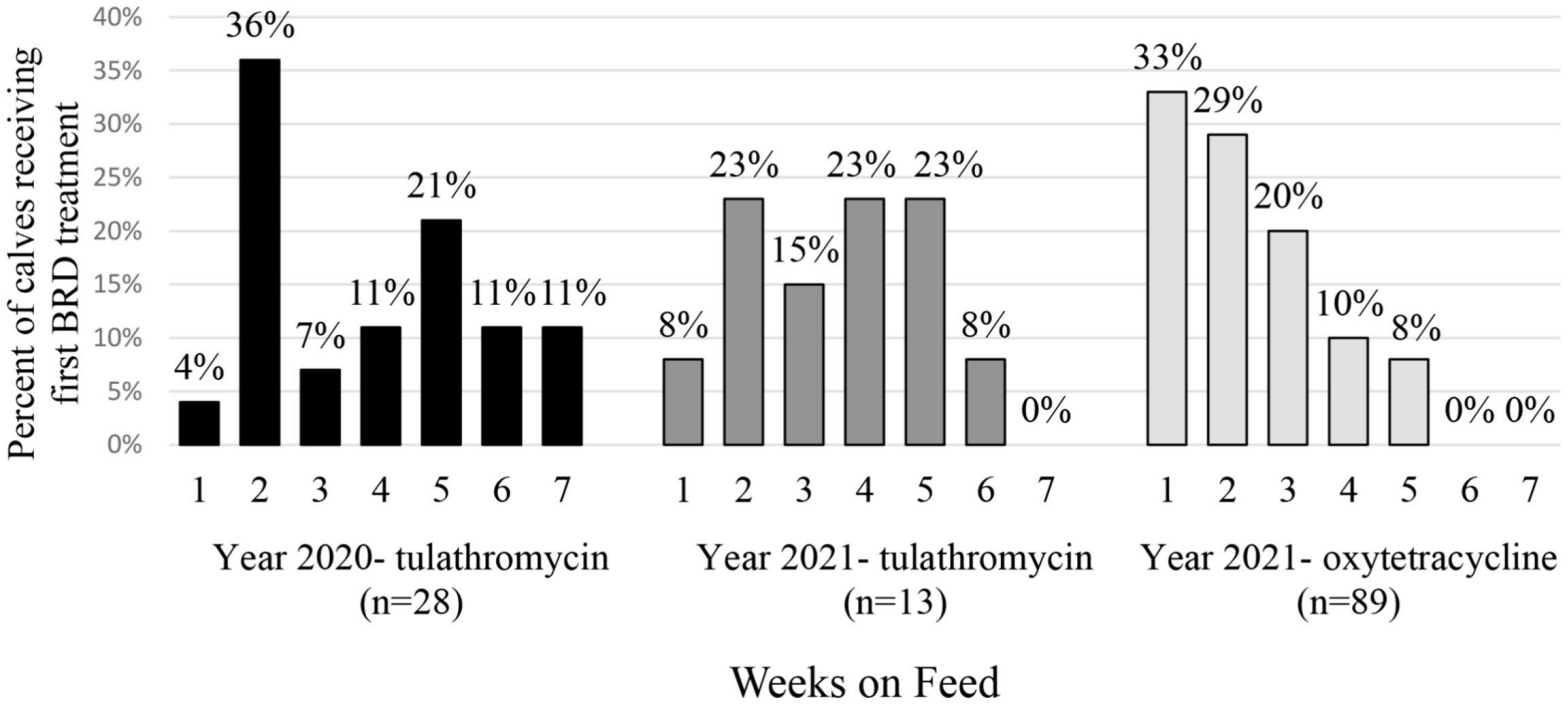
Percent (%) of calves receiving first treatment for BRD by weeks on feed. Calves are separated by sampling year and metaphylactic antimicrobial administered.

Treatment for BRD also varied by weeks on feed, year and metaphylaxis (Figure 1). In 2020 following tulathromycin metaphylaxis, most calves were treated the first time for BRD during week 2 (36% of total sick calves). In 2021, most of the calves with tulathromycin metaphylaxis were treated for the first time for BRD in weeks 2, 4, and 5 (23% each). However, for calves receiving oxytetracycline, BRD treatment risk was highest in week 1 (33% of calves). Regardless of year or type of metaphylaxis, very few calves required BRD treatment by week 6 or 7 on feed (Figure 1).

Half of the first treatments (65/130) for BRD were reported at or before the second sample (13DOF). The median DOF when calves received first BRD treatment was 24 days (IQR = 19 days) in those pens that received tulathromycin metaphylaxis, and 11 days (IQR = 13 days) for calves from pens that received oxytetracycline metaphylaxis (*p <* 0.001). At treatment, 82% of calves were given a BRD clinical score of 2, 17% a score of 3, and 2% a score of 1.

At the time of first treatment for BRD, bacteria of interest were recovered in 88% of calves. Regardless of year and type of metaphylaxis, *M. haemolytica* was the most common bacteria isolated from calves at first treatment for BRD (Table 3). In 2021, the second-most common type of bacteria was *P. multocida*; however, in 2020, *P. multocida* and *H. somni* were recovered in similar proportions. Type of metaphylaxis was not associated with the likelihood of recovering *M. haemolytica*, *P. multocida*, or *H. somni* at the time of BRD treatment (Table 4; Supplementary Table S6). *M. haemolytica* monoculture was the most common isolation pattern recovered in 35% of BRD-treated calves (Table 5), followed by co-isolation of *M. haemolytica* and *P. multocida* in 15% of BRD-treated calves. All three bacteria were recovered from 11% of BRD-treated calves that received tulathromycin in 2020, 4% in 2021 oxytetracycline-treated calves, and 0% in 2021 tulathromycin-treated calves (Table 5). Only 12% of BRD-treated calves had no bacteria of interest recovered from DNP samples at time of BRD treatment (Table 5).

**Table 3:** Crude number (%) of bacterial isolates recovered from calves sampled at time of first treatment for BRD (*n* = 1,599 calves at risk, *N* = 16 pens).

Year	Injectable metaphylaxis	Bacteria	Number (%) isolates recovered
2020	Tulathromycin *n* = 28 calves	*M. haemolytica*	21 (75%)
		*P. multocida*	8 (29%)
		*H. somni*	9 (32%)
2021	Tulathromycin *n* = 13 calves	*M. haemolytica*	9 (69%)
		*P. multocida*	4 (31%)
		*H. somni*	3 (23%)
	Oxytetracycline *n* = 89 calves	*M. haemolytica*	54 (61%)
		*P. multocida*	36 (40%)
		*H. somni*	25 (28%)

**Table 4:** Associations between study year, metaphylaxis antimicrobial administered and bacterial recovery from calves at the time of risk of first BRD treatment accounting for days on feed and clustering by pen.

			OR	95% CI		*p-*value
Bacteria	Year	Metaphylaxis		Lower	Upper	
*M. haemolytica*	2020	Tulathromycin	Reference category			
	2021	Tulathromycin	0.8	0.1	4.2	0.77
	2021	Oxytetracycline	0.5	0.1	2.3	0.40
*P. multocida*	2020	Tulathromycin	Reference category			
	2021	Tulathromycin	1.0	0.1	10.5	0.98
	2021	Oxytetracycline	1.8	0.5	7.1	0.39
*H. somni*	2020	Tulathromycin	Reference category			
	2021	Tulathromycin	0.6	0.2	2.3	0.49
	2021	Oxytetracycline	0.8	0.3	2.0	0.67

*n* = 130 calves treated for BRD (*N* = 16 pens). OR, odds ratio; 95% CI, 95% confidence interval.

**Table 5:** Co-isolation patterns of bacteria^*^ recovered from calves at time of first BRD treatment (*N* = 16 pens).

			Bacterial isolation pattern							
Year	Metaphylaxis	Number Calves	Neg. Culture	MH	PM	HS	MH + PM	MH + HS	PM + HS	MH + PM + HS
2020	Tulathromycin	28	6 (21%)	9 (32%)	0 (0%)	0 (0%)	4 (14%)	5 (18%)	1 (4%)	3 (11%)
2021	Tulathromycin	13	1 (8%)	6 (46%)	1 (8%)	1 (8%)	2 (15%)	1 (8%)	1 (8%)	0 (0%)
2021	Oxytetracycline	89	9 (10%)	31 (35%)	11 (12%)	7 (8%)	13 (15%)	6 (7%)	8 (9%)	4 (4%)
**All years**	**All groups**	130	16 (12%)	46 (35%)	12 (9%)	8 (6%)	19 (15%)	12 (9%)	10 (8%)	7 (5%)

*M. haemolytica, MH; *P. multocida*, PM; *H. somni*, HS.

### 3.2 Bacterial recovery from BRD treated calves at 1DOF, 13DOF, and time of first BRD treatment

**Figure 2:**
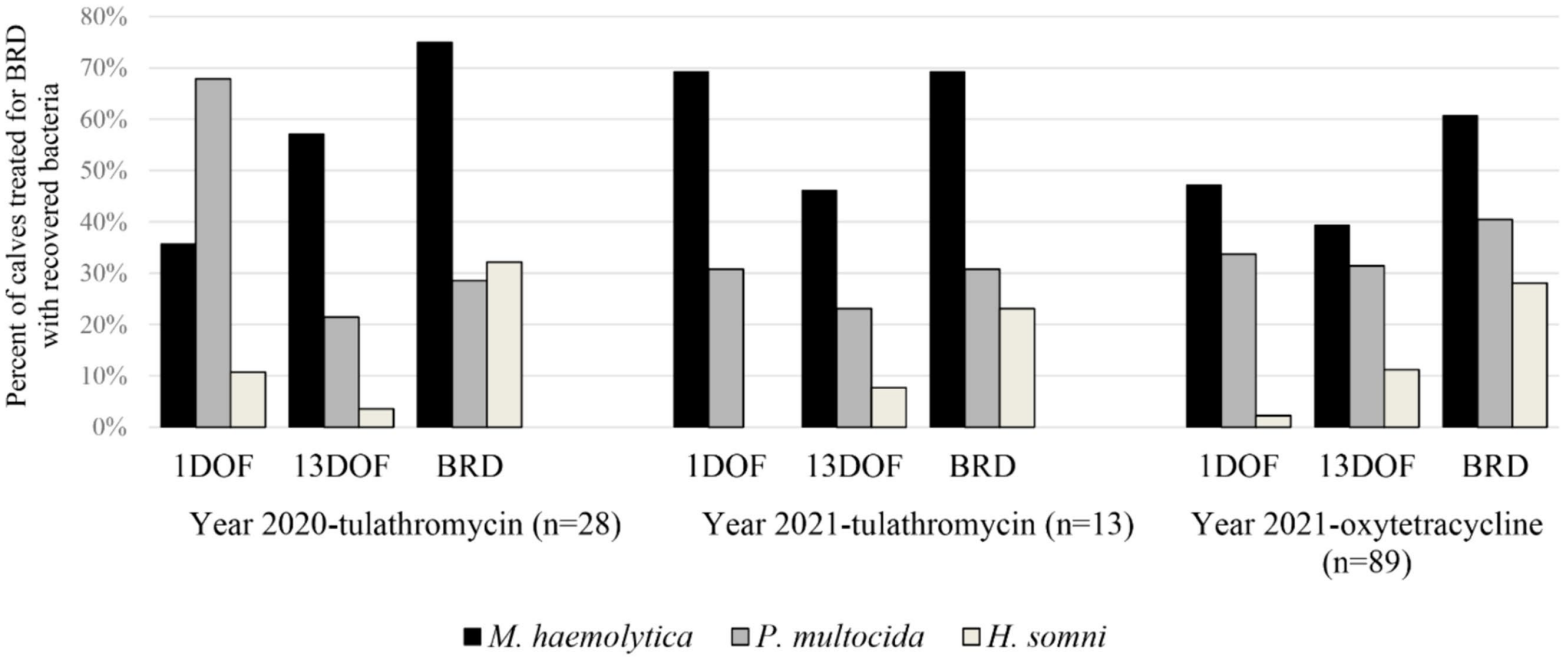
Percent (%) of calves receiving first treatment for BRD by sample collection time with recovered *Mannheimia haemolytica*, *Pasteurella multocida*, and *Histophilus somni*. Calves are separated by sampling year and metaphylactic antimicrobial administered.

Generally, bacteria of interest were recovered in greater proportions from calves at time of BRD treatment than at 1DOF or 13DOF (Figure 2). *M. haemolytica* was the most common bacteria recovered at time of BRD treatment while the most common bacteria recovered at 1DOF or 13DOF was either *M. haemolytica* or *P. multocida*. *H. somni* was more frequently recovered at time of BRD treatment than at 1DOF or 13DOF. Detailed information on differences in bacterial recovery and AMR in calves at 1DOF and 13DOF was previously reported (21).

### 3.3 Antimicrobial-resistant bacterial isolates recovered from calves at time of first BRD treatment

At the time of first BRD treatment, bacteria recovered from most calves (70%, 91/130) were pan susceptible to the tested antimicrobials. Twelve calves from 2020 that had received tulathromycin metaphylaxis had at least one resistant isolate at first BRD treatment. In 2021, two calves receiving tulathromycin and 25 calves receiving oxytetracycline at arrival also had at least one resistant isolate at first BRD treatment. The likelihood of having a pathogen with resistance to any type of antimicrobial at BRD treatment did not differ based on year or metaphylaxis (2021 tulathromycin vs. 2020 tulathromycin, *p =* 0.27; 2021 oxytetracycline vs. 2020 tulathromycin, *p =* 0.28; 2021 oxytetracycline vs. 2021 tulathromycin, *p =* 0.56).

The most common type of resistance at first BRD treatment was to tetracycline (18%, 24/130 BRD calves) (Table 6); however, all but one resistant isolate was from 2021. Tetracycline resistance was observed in only one *M. haemolytica* isolate from a 2020 tulathromycin-treated calf. Twenty-three calves that received oxytetracycline at arrival in 2021 had either *P. multocida* or *H. somni* isolates with tetracycline resistance at first BRD treatment. Tetracycline resistance at BRD treatment was, therefore, more likely from calves that received oxytetracycline on arrival than tulathromycin (OR = 15, 95% CI = 1.8–128, *p =* 0.01).

**Table 6:** Number (%) of calves at time of first BRD treatment with bacteria interpreted as resistant to select antimicrobials based on CLSI breakpoints (*N* = 16 pens).

Year	Resistant isolates based on available CLSI breakpoints^*^										
Metaphylaxis^**^	Bacteria^***^	AMP	PEN	TIO	DANO	SPECT	TET	GAM	TILD	TILM	TULA
2020 tulathromycin *n* = 28 calves	MH	1 (3.6%)	1 (3.6%)	1 (3.6%)	0	0	1 (3.6%)	9 (32%)	0	1 (3.6%)	6 (21%)
	PM	1 (3.6%)	0	0	0	0	0	0	0	NI	0
	HS	0	0	0	NI	0	0	0	0	NI	0
2021 tulathromycin *n* = 13 calves	MH	0	0	0	0	0	0	2 (15%)	2 (15%)	2 (15%)	2 (15%)
	PM	0	0	0	0	0	0	0	0	NI	0
	HS	0	0	0	NI	0	0	0	0	NI	0
2021 oxytetracycline *n* = 89 calves	MH	0	1 (1.1%)	0	0	0	0	0	0	0	0
	PM	1 (1.1%)	0	0	0	15 (17%)	15 (17%)	0	0	NI	0
	HS	0	1 (1.1%)	0	NI	0	8 (9.0%)	0	0	NI	0
Total *n* = 130 calves		3 (2.3%)	3 (2.3%)	1 (0.7%)	0 (0%)	15 (12%)	24 (18%)	11 (8.5%)	2 (1.5%)	3 (2.3%)	8 (6.2%)

^*^Antimicrobials for which isolates were tested: AMP, ampicillin; PEN, penicillin; TIO, ceftiofur; DANO, danofloxacin; SPECT, spectinomycin; TET, tetracycline; GAM, gamithromycin; TILD, tildipirosin; TILM, tilmicosin; TULA, tulathromycin. ^**^ Metaphylaxis administered at arrival. ^***^ Bacteria: MH, *M. haemolytica*; PM, *P. multocida*; HS, *H. somni*. NI = not interpretable. There were no samples from calves with bacteria resistant to enrofloxacin or florfenicol.

All of the calves treated for BRD with macrolide-resistant *M. haemolytica* (8%, 11/130) had received tulathromycin metaphylaxis, nine calves from 2020 and two calves from 2021 (Table 6). Resistance was most common to the 15-membered ring macrolides gamithromycin or tulathromycin.

Only two calves from the pens receiving tulathromycin in 2021 had any resistant isolates recovered at BRD treatment (Table 6). However, these were the only treated calves with isolates resistant to all macrolides of interest: gamithromycin, tulathromycin, tildipirosin, and tilmicosin.

At the time of first BRD treatment, no calves had isolates with resistance to danofloxacin, enrofloxacin, or florfenicol (Table 6). Ceftiofur-resistant *M. haemolytica* was observed in one BRD-treated calf sampled in 2020. Three treated calves had bacteria resistant to ampicillin or penicillin; one received oxytetracycline metaphylaxis and two were from tulathromycin-treated pens. Antimicrobial resistant *H. somni* were only observed in 2021 oxytetracycline-treated calves; one calf (1%) had a penicillin-resistant *H. somni* isolate and eight calves (9%) had tetracycline-resistant *H. somni* isolates.

Multi-class resistant *P. multocida* was identified in one calf from the tulathromycin cohort in 2021 at arrival and again at the time of first treatment. Resistance was reported for ampicillin, tetracycline, and spectinomycin.

### 3.4 Predicting likelihood of first BRD treatment given culture and susceptibility for calves at 1DOF and 13DOF

For the 1,599 calves sampled at 1DOF, those with any tetracycline-resistant bacteria of interest were more likely to be treated for BRD at or before 13DOF compared to calves without tetracycline-resistant bacteria of interest (OR = 1.8, *p =* 0.021; Table 7) (AUC = 0.77). No other C/S results at 1DOF were associated with an increased likelihood of a calf requiring first BRD treatment during the first 45DOF (*n* = 130 total calves treated for BRD) (Table 7; Supplementary Table S7).

**Table 7:** Associations between isolation of various bacteria from individual calves at 1DOF (*n* = 1,599 calves) or 13DOF (*n* = 1,596 calves) and treatment for BRD.

Risk factor: calf culture positive	OR	95% CI		*p-*value
		Upper	Lower	
**Calf culture positive at 1DOF (*n* = 1,599)**	**Outcome: BRD** ≤ **13DOF**			
*M. haemolytica*	0.9	0.7	1.3	0.62
*P. multocida*	1.07	0.7	1.7	0.77
*H. somni*	0.97	0.4	2.1	0.93
Any bacteria with AMR	1.8	0.9	3.6	0.09
Any bacteria with macrolide resistance*	1.4	0	6.8	0.61
**Any bacteria with tetracycline resistance**	**1.8**	**1.1**	**2.8**	**0.021**
**Calf culture positive at 1DOF (*n* = 1,599)**	**Outcome: BRD** ≤ **45DOF**			
*M. haemolytica*	1.1	0.9	1.4	0.46
*P. multocida*	0.9	0.6	1.3	0.65
*H. somni*	0.7	0.3	1.4	0.27
Any bacteria with AMR	0.9	0.4	2.0	0.85
Any bacteria with macrolide resistance*	0.7	0	3.2	0.36
Any bacteria with tetracycline resistance	0.8	0.5	1.5	0.55
**Calf culture positive at 13DOF (n = 1,596)**	**Outcome: BRD** > **13DOF**			
***M. haemolytica***	**2.1**	**1.3**	**3.3**	**0.004**
***P. multocida***	**2.2**	**1.1**	**4.3**	**0.018**
***H. somni***	**1.6**	**1.1**	**2.4**	**0.028**
Any bacteria with AMR	1.3	0.8	2.1	0.32
Any bacteria with macrolide resistance	1.2	0.5	2.7	0.73
Any bacteria with tetracycline resistance	1.2	0.6	2.2	0.66

Analysis accounted for year and injectable metaphylaxis administered and adjusts for clustering by pen (*N* = 16 pens). OR, odds ratio; 95% CI, 95% confidence interval. *Exact logistic regression (SAS^®^ version 9.4, Cary, NC, United States). See details for other fixed effects and measures of model fit in Supplementary Table S7.

In contrast, several bacteria of interest recovered from calves at 13DOF (*n* = 1,596 calves sampled) were associated with BRD treatment after 13DOF (Table 7). The recovery of *M. haemolytica*, *P. multocida*, or *H. somni* at 13DOF was associated with approximately twice the likelihood of a calf requiring BRD treatment after 13DOF (Table 7). Antimicrobial susceptibility results at 13DOF were not associated with an increased risk of BRD treatment between 14DOF and 45DOF (Table 7).

### 3.5 Associations between culture and susceptibility for calves at 1DOF and culture and susceptibility for calves at time of first BRD treatment

For calves receiving first treatment for BRD at or before 13DOF, only three calves had tetracycline resistance in bacteria of interest at 1DOF. For BRD-treated calves, those calves with tetracycline-resistant bacteria at 1DOF were more likely to retain tetracycline-resistant bacteria at BRD treatment, if treated for BRD within 13DOF (OR = 12, *p =* 0.02; Table 8; Supplementary Table S8) (AUC = 0.99). Meanwhile, calves with tetracycline bacteria recovered at 13DOF were less likely to have resistance if treated within the next 30 days (OR = 0.08, *p =* 0.02; Table 8). However, the AUC for this model was 0, indicating no predictive performance, likely due to the limited number of calves with tetracycline resistance. No BRD treated calves had macrolide-resistant isolates of interest recovered at 1DOF. No other significant associations were found between positive C/S results at 1DOF and the same C/S results from calves at time of first BRD treatment (Table 8).

**Table 8:** For calves treated for BRD within 13DOF (*n* = 65) and all calves treated for BRD within 45DOF (*n* = 130), associations between deep nasopharyngeal culture and susceptibility (C/S) test results for calves at 1DOF and likelihood of corresponding C/S results at time of first BRD treatment.

Risk factor: calf C/S at 1DOF	Outcome: calf C/S at BRD treatment ≤ 13DOF	OR	95% CI		*p-*value
			Lower	Upper	
M. haemolytica*	*M. haemolytica*	1.1	0.6	2.2	0.78
*P. multocida*	*P. multocida*	1.9	0.6	5.9	0.26
H. somni*	*H. somni*	9.8	0.9	108	0.06
Any bacteria with AMR*	Any bacteria with AMR	3.0	0.6	15	0.18
Any bacteria with macrolide resistance**	Any bacteria with macrolide resistance	Not estimable			
Any bacteria with tetracycline resistance***	**Any bacteria with tetracycline resistance**	**12**	**1.7**	**∞**	**0.018**

Risk factor: calf C/S at 1DOF	Outcome: calf C/S at BRD treatment ≤ 45DOF	OR	95% CI		*p-*value
			Lower	Upper	
*M. haemolytica*	*M. haemolytica*	1.1	0.6	2.2	0.74
*P. multocida*	*P. multocida*	1.5	0.8	2.8	0.27
*H. somni*	*H. somni*	2.0	0.3	15	0.50
Any bacteria with AMR	Any bacteria with AMR	2.1	0.6	7.5	0.25
Any bacteria with macrolide resistance**	Any bacteria with macrolide resistance	Not estimable			
Any bacteria with tetracycline resistance***	**Any bacteria with tetracycline resistance**	**0.08**	**0**	**0.57**	**0.016**

Analysis accounted for year, injectable metaphylaxis administered, and DOF at time of BRD treatment, and adjusted for clustering by pen (*N* = 16 pens). OR, odds ratio; 95% CI, 95% confidence interval. *Unconditional GEE accounting for clustering by pen. **No samples from calves treated for BRD had bacteria with macrolide resistance at 1DOF. *** Exact logistic regression completed for tetracycline resistance: 3 sick calves with tetracycline resistance at 1DOF (SAS^®^ version 9.4, Cary, NC, United States) adjusted for year and metaphylaxis. See details for other fixed effects and measures of model fit in Supplementary Table S8.

### 3.6 Associations between culture and susceptibility results for calves at 13DOF and culture and susceptibility results for calves at time of first BRD treatment after 13DOF

Of the three bacterial pathogens examined, only calves with *P. multocida* recovered at 13DOF were also more likely to have *P. multocida* at time of BRD treatment (OR = 4.4, *p =* 0.02; Table 9; Supplementary Table S9) (AUC = 0.98). Calves with bacteria of interest with any AMR at 13DOF were more likely to have bacteria of interest with AMR at BRD treatment (OR = 4.9, *p =* 0.04; Table 9) (AUC = 0.99). Models including a fixed effect for year and metaphylaxis for analysis of AMR for macrolides and tetracyclines failed to converge. However, in unconditional models accounting only for clustering by pen, macrolide resistance and tetracycline resistance detected at 13DOF were both associated with subsequent detection of corresponding AMR at BRD treatment (Table 9).

**Table 9:** For calves treated for BRD after 13DOF (*n* = 65), associations between deep nasopharyngeal culture and susceptibility (C/S) test results at 13DOF and likelihood of corresponding C/S results for calf at time of BRD treatment.

			95% CI		
Risk factor: calf culture positive at 13DOF	Outcome: calf C/S result at BRD treatment	OR	Lower	Upper	*p-*value
*M. haemolytica*	*M. haemolytica*	2.8	0.7	11	0.15
*P. multocida*	*P. multocida*	**4.4**	**1.3**	**15**	**0.019**
*H. somni*	*H. somni*	2.5	0.8	8.3	0.13
**Any bacteria with AMR**	**Any bacteria with AMR**	**4.9**	**1.1**	**21**	**0.036**
Any bacteria with macrolide resistance*	**Any bacteria with macrolide resistance**	**9.8**	**1.2**	**77**	**0.030**
Any bacteria with tetracycline resistance*	**Any bacteria with tetracycline resistance**	**2.9**	**1.8**	**4.6**	**< 0.001**

Analysis accounted for year and injectable metaphylaxis administered, and DOF at time of BRD treatment, and adjust for clustering by pen-cohort unless otherwise noted (*N* = 16 pens). OR, odds ratio; 95% CI, 95% confidence interval. *Unconditional GEE logistic regression reported: no calves with BRD from 2021/oxytetracycline cohorts had macrolide resistance at time of BRD treatment and no calves from 2021/tulathromycin cohorts had tetracycline resistance at time of BRD treatment. See details for other fixed effects and measures of model fit in Supplementary Table S9.

### 3.7 Likelihood of calves from a specific pen requiring first treatment for BRD given pen-level prevalence of bacteria and antimicrobial susceptibility at 1DOF and 13DOF

No C/S results at 1 or 13DOF were associated with an increase in BRD treatment during the study (Table 10; Supplementary Table S10). The only significant association was between an increase in pen-level prevalence of *H. somni* at 1DOF and a decrease in the odds of receiving BRD treatment within 45DOF (*p* = 0.02; Table 10) (AUC = 0.76). Further exploration into bacterial isolation patterns revealed a negative association between recovery of *M. haemolytica* and *P. multocida* at 1DOF (Spearman’s ρ = −0.55; *p =* 0.02). No significant correlations were noted between *M. haemolytica* and *H. somni*; however, some pens had no calves with detected *H. somni* at 1DOF.

**Table 10:** Associations between pen-level prevalence of deep nasopharyngeal culture and susceptibility (C/S) results and pen-level risk of BRD.

Risk factor: increase in pen-level C/S results	Outcome: pen BRD prevalence		95% CI		
		OR	Lower	Upper	*p-*value
**Prevalence at 1DOF**	**BRD** ≤ **13DOF**				
*M. haemolytica* (5%)		1.05	0.98	1.1	0.16
*P. multocida* (5%)		1.03	0.97	1.1	0.28
*H. somni* (5%)		0.96	0.8	1.2	0.68
Any AMR (5%)		1.02	0.98	1.1	0.46
Macrolide resistance (5%)		1.03	0.97	1.1	0.27
Tetracycline resistance (5%)		0.99	0.97	1.00	0.08
**Prevalence at 1DOF**	**BRD** ≤ **45DOF**				
*M. haemolytica* (5%)		1.0	0.8	1.3	0.76
*P. multocida* (5%)		0.94	0.9	1.0	0.11
***H. somni* (5%)**		**0.7**	**0.5**	**0.9**	**0.02**
Any AMR (5%)		0.7	0.4	1.3	0.31
Macrolide resistance (5%)		0.4	0.04	4.7	0.48
Tetracycline resistance (5%)		0.9	0.51	1.5	0.67
**Prevalence at 13DOF**	**BRD** > **13DOF**				
*M. haemolytica* (5%)		0.9	0.6	1.2	0.51
*P. multocida* (5%)		0.9	0.8	1.1	0.24
*H. somni* (5%)		0.6	0.4	1.1	0.10
Any AMR (5%)		0.9	0.4	1.9	0.72
Macrolide resistance (5%)		0.3	0.03	2.4	0.23
Tetracycline resistance (5%)		1.2	0.6	2.3	0.56

Analysis adjusted for year and metaphylaxis as well as accounting for clustering by pen cohort. *n* = 1,599 calves at 1DOF and 1,596 calves at 13DOF (*N* = 16 pens). OR, odds ratio; 95% CI, 95% confidence interval.

### 3.8 Likelihood of culture and susceptibility results from any sick calf from a specific pen at time of first BRD treatment given pen-level prevalence of bacteria at 1DOF and 13DOF

An increasing pen-level prevalence of macrolide resistance, tetracycline resistance, or any AMR at 1DOF was associated with an increased risk of these same AMR patterns in isolates from calves treated for BRD within the first 13DOF (Table 11; Supplementary Table S11). Namely, for every 5% increase in pen-level prevalence of macrolide resistance at 1DOF, a calf within the pen would have 975 times greater odds of having macrolide resistant bacteria recovered at BRD treatment within the next 13 days on feed (*p* ≤ 0.001) (AUC = 0.81). For every 5% increase in pen-level prevalence of tetracycline resistance at 1DOF, a calf within the pen would have 3.4 times greater odds of having tetracycline resistant bacteria recovered if receiving BRD treatment within 13DOF (*p* = 0.003) (AUC = 0.64).

**Table 11:** Associations between pen-level prevalence of deep nasopharyngeal culture and susceptibility (C/S) results at 1DOF and 13DOF and pen C/S prevalence at time of BRD treatment.

Risk factor: increase in	Outcome: pen prevalence		95% CI		
Pen-level C/S prevalence	C/S at BRD treatment	OR	Lower	Upper	*p-*value
**Prevalence at 1DOF**	**BRD** ≤ **13DOF**				
*M. haemolytica* (5%)	*M. haemolytica*	1.2	0.8	1.9	0.40
*P. multocida* (5%)	*P. multocida*	**0.5**	**0.2**	**1.0**	**0.04**
*H. somni* (5%)	*H. somni*	1.4	0.7	2.7	0.37
Any AMR (5%)*	**Any AMR**	**5.6**	**2.1**	**15.3**	**0.0008**
Macrolide resistance (5%)*	**Macrolide resistance**	**975**	**33**	**29,439**	**<0.0001**
Tetracycline resistance (5%)*	**Tetracycline resistance**	**3.4**	**1.5**	**7.6**	**0.003**
Prevalence at 1DOF	BRD ≤ 45DOF				
*M. haemolytica* (50%)	*M. haemolytica*	1.3	0.9	1.8	0.19
*P. multocida* (50%)	*P. multocida*	0.7	0.5	1.0	0.067
*H. somni* (50%)	*H. somni*	1.1	0.6	2.1	0.67
Any AMR (50%)	Any AMR	1.8	0.3	9.7	0.49
Macrolide resistance (5%)*	**Macrolide resistance**	**22**	**1.8**	**263**	**0.02**
Tetracycline resistance (5%)*	**Tetracycline resistance**	**11**	**5.4**	**23**	**<0.0001**
Prevalence at 13DOF	BRD > 13DOF				
*M. haemolytica* (5%)	*M. haemolytica*	**1.4**	**1.2**	**1.7**	**<0.0001**
*P. multocida* (5%)	*P. multocida*	1.2	0.9	1.6	0.26
*H. somni* (5%)	*H. somni*	1.1	0.7	1.6	0.69
**Any AMR (5%)**	**Any AMR**	**1.7**	**1.4**	**2.1**	**<0.0001**
**Macrolide resistance (5%)**	**Macrolide resistance**	**1.3**	**1.2**	**1.5**	**<0.0001**
**Tetracycline resistance (5%)**	**Tetracycline resistance**	**1.8**	**1.4**	**2.4**	**<0.0001**

Analysis adjusted for year and metaphylaxis as well as accounting for clustering by pen cohort. *n* = 1,599 calves at 1DOF and 1,596 calves at 13DOF (*N* = 16 pens). OR, odds ratio; 95% CI, 95% confidence interval. **Unconditional GEE logistic regression reported: No calves with BRD from 2021/oxytetracycline cohorts had macrolide resistance at time of BRD treatment and no calves from 2021/tulathromycin cohorts had tetracycline resistance at time of BRD treatment.

Increasing pen-level prevalence of tetracycline (AUC = 0.77) or macrolide resistance (AUC = 0.63) at 1DOF was also associated with subsequently observing these C/S patterns in isolates from calves in that pen treated for BRD at any time during the 45-day trial (Table 11).

Finally, an increased pen-level prevalence of *M. haemolytica* (AUC = 0.76), tetracycline (AUC = 0.85), macrolide (AUC = 0.94), or any AMR resistance (AUC = 0.89) at 13DOF was associated with these C/S patterns being present in isolates from calves treated for BRD between 14DOF and 45DOF (Table 11). Specifically, for a 5% increase in pen-level prevalence of macrolide resistance at 13DOF, the odds of a calf within the pen having macrolide resistance at time of BRD treatment increased by 1.3 times (*p* ≤ 0.001). Given that this was a linear association on the log odds scale, the odds of macrolide resistance at the time of BRD treatment would be expected to increase 3.1 times (=1.3^4^) for a 20% difference between in pen prevalence of macrolide AMR at 13DOF and 9.8 times (=1.3^8^) at the time of BRD treatment for a 40% difference between pens at 13DOF.

The association between pen prevalence of tetracycline resistance at 13DOF and the odds of tetracycline resistance if a calf was treated after 13DOF was also significant (Table 11). For a 5% increase in pen-level prevalence of tetracycline resistance at 13DOF, the odds of a calf within the pen having tetracycline resistance at time of BRD treatment increased by 1.8 times (*p* ≤ 0.001).

### 3.9 Associations between calf culture and susceptibility results near 36DOF given calf- and pen-level prevalence of bacteria at 1DOF and 13DOF

A subset of calves from each pen were sampled at a third time point near 36DOF (n = 310). Complete results of regression models as outlined in Supplementary Table S1 are available in Supplementary Tables S2–S5. The recovery of isolates from calves with tetracycline resistance at 1DOF was associated with an increased risk of subsequent tetracycline resistance at 36DOF (Supplementary Table S2). Calves from which *P. multocida* were recovered at either 1DOF or 13DOF were also at increased risk of having *P. multocida* at the third sampling time (Supplementary Tables S2, S3).

Consistent with previous predictive C/S patterns observed at 13DOF, an increasing pen-level prevalence of *P. multocida*, resistance to tetracyclines, resistance to macrolides, or overall increasing presence of AMR were all associated with a greater likelihood of these culture patterns being subsequently observed in isolates from calves near 36DOF (Supplementary Table S4).

### 3.10 Summary of model outcomes

Calf C/S results associated with the risk of BRD and future C/S patterns are summarized in Table 12. Pen-level prevalence of variables at 1DOF and 13DOF that were associated with increasing risk of BRD and future C/S patterns are summarized in Table 13. Results for models predicting status at 36DOF are summarized in Supplementary Table S5.

**Table 12:** Summary of calf-level culture and susceptibility (C/S) results at 1DOF and 13DOF and their association with individual calf risk of BRD and likelihood of subsequent C/S patterns at time of treatment.

BRD risk given C/S results near arrival		
Risk factor	Outcome	Significant risk factors for increasing risk*
C/S results at 1DOF	Risk of BRD ≤ 13DOF	Tetracycline resistance
C/S results at 1DOF	Risk of BRD ≤ 45 DOF	None
C/S results at 13DOF	Risk of BRD > 13DOF	*M. haemolytica*, *P. multocida*, *H. somni*

Associations between C/S results at time of BRD treatment given C/S results near arrival		
Risk factor	Outcome	Significant risk factors for increasing risk**
C/S results at 1DOF	C/S results at BRD treatment ≤13DOF	Tetracycline resistance
C/S results at 1DOF	C/S results at BRD treatment ≤45DOF	None
C/S results at 13DOF	C/S results at BRD treatment >13DOF	*P. multocida,* any bacteria with AMR, macrolide, or tetracycline resistance

* Table 7, ** Tables 8, 9.

**Table 13:** Summary of pen-level prevalence of culture and susceptibility (C/S) results at 1DOF and 13DOF associated with pen-level risk of BRD and C/S results at time of BRD treatment.

BRD risk given pen-level C/S prevalence near arrival		
Risk factor	Outcome	Significant risk factors for increasing risk*
Pen prevalence at 1DOF	Risk of BRD ≤ 13DOF	None
Pen prevalence at 1DOF	Risk of BRD ≤ 45 DOF	None
Pen prevalence at 13 DOF	Risk of BRD > 13 DOF	None

Associations between C/S results at time of BRD treatment given pen-level C/S prevalence near arrival		
Risk factor	Outcome	Significant risk factors for increasing risk**
Pen prevalence at 1DOF	C/S results at BRD treatment ≤13DOF	Any AMR, macrolide, or tetracycline resistance
Pen prevalence at 1DOF	C/S results at BRD treatment ≤45 DOF	Macrolide or tetracycline resistance
Pen prevalence at 13DOF	C/S results at BRD treatment >13DOF	*M. haemolytica,* any AMR, macrolide, or tetracycline resistance

* Table 10, ** Table 11.

## 4 Discussion

Evidence-based antimicrobial treatment protocols are recommended components of antimicrobial stewardship efforts. However, no practical laboratory-based guidelines are currently available to inform antimicrobial selection and use in feedlot calves with BRD. Chute-side diagnostics for use in individual animal treatment plans might be an eventual goal but, for now, better strategies are needed using the current and available diagnostic tools. One of the biggest drawbacks of current tools is the time required to both deliver the samples to the diagnostic laboratory and for the laboratory tests themselves. This study examined how calf- and pen-level C/S data obtained in the first two weeks on feed could aid in predicting future risk of BRD as well as the AMR profile of calves at time of BRD diagnosis. The results help to address the question of whether samples from individuals or pens collected before the expected peak of BRD cases could inform treatment strategies.

Examples of both individual calf C/S results and pen prevalence of C/S results obtained within two weeks of feedlot arrival were associated with the risk of clinical BRD and AMR bacteria at BRD diagnosis. Most relevant to supporting prudent antimicrobial treatment choices was that an increasing pen-level prevalence of macrolide- or tetracycline-resistant bacteria at both 1DOF and 13DOF was associated with an increased risk of these resistance profiles at BRD treatment. Resistance in calves sampled at 13DOF was similarly associated with resistance in samples collected near 36DOF. While resistance in samples from individual calves at 13DOF was also associated with resistance at the time of treatment, only tetracycline resistance at 1DOF was associated with subsequent resistance at treatment.

Findings from this study suggest a calf’s risk of BRD and C/S results at the time of treatment are influenced by the pen-level prevalence of bacteria and AMR, as well as the prior C/S results of the individual animal. As feedlot cattle are managed as groups, pen mates have the potential to influence calf health and risk of acquiring AMR bacteria. This was demonstrated in a study describing horizontal transmission of BRD among pen mates, where feedlot calves were fitted with real-time location system transmitter tags to observe comingling and BRD status within a pen (18). Healthy animals were more likely to develop BRD when in close contact with diseased cattle, presumably shedding BRD pathogens, suggesting the communicable nature of these pathogens (18). Additionally, another study used whole genome sequencing to report evidence of horizontal transmission of *M. haemolytica* strains between arrival and revaccination in a group of stocker cattle (19). The *M. haemolytica* isolates collected at arrival were classified in four different clades, while all isolates from revaccination were from one clade (19). In a further example, dissemination of a tetracycline-resistant *P. multocida* clone was observed within three weeks of feedlot arrival (20). However, contrasting results were reported by Woolums et al. (25), who found differing clones of *M. haemolytica* shed by stocker cattle sampled multiple times over a 21-day study.

The extent of transmission between calves within pens can also be inferred by examination of clustering of culture results within pens. For example, pen-level clustering, reported as the intraclass correlation coefficient (ICC) in Abi Younes et al. (21), substantially increased with DOF for *M. haemolytica* with macrolide or tetracycline resistance. The ICC of pens with these resistance profiles was near negligible at 1DOF (0.1%), indicating very little clustering of observations within newly formed cohorts at arrival. However, the ICC of AMR bacteria within pens increased to over 70% by 13DOF and supports the findings of the current analysis. Increased pen-level recovery of AMR at 13DOF was associated with an increased likelihood that calves sampled from the pen at BRD treatment, or later in the feeding period (near 36DOF), would have the same resistance patterns.

Together, these examples highlight the connections among calves within a pen and the importance of considering group health and the dynamics of microbial transmission between calves when managing BRD and antimicrobial resistance risk. Pen-level sampling strategies could inform antimicrobial protocols by providing evidence to limit the use of antimicrobials for which there is substantial proof of resistance, thereby supporting antimicrobial stewardship efforts. This would provide further benefits by potentially reducing the number of treatment failures associated with AMR as well as limiting further selection for AMR. Calves that fail to respond to first lines of therapy are traditionally empirically retreated using a different antimicrobial class, which may or may not be the most efficacious choice. Regardless, laboratory-based evidence would provide support treatment protocols.

Most recent studies evaluating bacterial pathogens in feedlot cattle have focused on differences in the recovery of bacterial pathogens between healthy and BRD-affected cattle (12, 14, 27–29), changes in bacterial recovery over time (10, 14, 30–32), or differences in risk of BRD between auction-derived and ranch-direct calves (20, 33). A few studies have examined whether DNP samples on arrival to the feedlot can predict the risk of BRD treatment (13, 15), but no reports to date have explored the prediction of subsequent antimicrobial susceptibility status at time of treatment. This investigation adds to the literature with 1,599 auction-derived, mixed-origin calves sampled at feedlot arrival, again at 13DOF, and at first BRD treatment. Most uniquely, this study considered the added value of pen prevalence of C/S outcomes on both subsequent pen risk of BRD and the likelihood of C/S outcomes in calves with BRD.

The two distinct sampling time points of 1DOF and 13DOF were deliberately chosen relative to peak risk for health outcomes. Sampling at arrival was selected for its relative logistical convenience, leveraging established routine processing. Additionally, this timing provided the opportunity to acquire laboratory results before the conclusion of the post-metaphylactic interval associated with commonly used long-acting antimicrobials and therefore prior to when most calves would be eligible for first BRD treatment.

Sampling at 13DOF was chosen to coincide with the maximum post-metaphylactic interval of tulathromycin (34). An exploration of the prevalence of AMR *M. haemolytica* isolates recovered prior to and following tulathromycin metaphylaxis has been reported in stocker calves (25, 35, 36). This interval after arrival allowed for the opportunity to capture any changes in respiratory flora associated with metaphylaxis (17), commingling, changes in environment, stress, and other risk factors associated with feedlot placement (10, 28). Sampling at 13DOF was also sufficiently early in the feeding period so as to expect most BRD cases to occur after this date, particularly for high-risk calves where tulathromycin metaphylaxis was used on arrival (37). In a recent investigation of 25 commercial feedlots, 25% of first BRD treatments for late fall placed calves were reported by day 15, while 75% of cases occurred by 53DOF (38). The current study observed a median DOF of 24 days for BRD calves that received tulathromycin metaphylaxis suggesting that data collected at 13DOF could be available before most BRD cases. However, for oxytetracycline-treated calves it was only 11DOF. While additional studies would be necessary to identify optimal sampling times for metaphylaxis protocols with a shorter duration of action, products such as oxytetracycline are less likely to be used in calves at the highest risk for BRD.

Culture and susceptibility patterns of individual calves at 1DOF were not consistently associated with future risk of BRD. These results were not unexpected. The failure of results from 1DOF to predict later outcomes likely stems from the substantial shifts observed in the respiratory microbiota of calves between feedlot arrival and later stages in the feeding period (10, 12, 31, 39). Differences in bacterial recovery from 1DOF to 13DOF were also observed in the larger calf populations evaluated in this study where the prevalence of *M. haemolytica*, *P. multocida*, and *H. somni,* as well as AMR patterns, varied over time within pens, between pens, and between years and antimicrobial metaphylaxis used (21).

Other investigations comparing culture results at feedlot arrival to time of BRD treatment have also reported mixed findings (13, 15). McMullen et al. (14) examined nasopharyngeal and tracheal samples from both BRD-affected cattle and healthy controls, but were unable to identify consistent patterns in composition or diversity over time. In contrast, Taylor et al. (15) reported that in the 395 auction-derived cattle enrolled in their study and transported to the Oklahoma State University research feedlot, those from which *M. haemolytica* were recovered near or subsequent to feedlot arrival were more likely to require treatment for BRD. Notably, calves in Taylor et al. (15) did not receive metaphylaxis treatment. Similarly, Noyes et al. (13) observed commercial feedlot cattle with *M. haemolytica* detected on arrival to be at nearly twice the risk of developing BRD within 10 d. In their study, samples were collected from cattle at four different commercial feedlots, and high-risk cattle were administered parenteral macrolide or tetracycline antimicrobials based on individual feedlot protocols (13).

In contrast to the limited predictive utility of sampling cattle at 1DOF, C/S associations from sampling at 13DOF were more consistently informative. And from the perspective of informing future antimicrobial treatment options, the most clinically meaningful findings were that AMR patterns identified near two weeks on feed were associated with the same AMR patterns recovered at time of BRD treatment. As observed, both individual and pen-level models showed a positive association between antimicrobial resistance recovery at 13DOF and subsequent recovery at time of BRD treatment. To assess and compare the predictive performance of the different models, we calculated the AUC for the ROC for the logistic models. The AUCs were higher for more models using C/S results from 13DOF than those based on data from 1DOF.

The association between pen-level sampling results and culture/susceptibility (C/S) outcomes at the time of subsequent BRD treatment appear to be unique to this study. While individual calf-level models indicated that culture results obtained during the early feeding period could be associated with a calf’s later culture results particularly for samples collected at 13DOF, collecting samples from each individual calf in the pen to inform future treatment strategies for that calf is impractical—both financially and logistically. While the pen-level prevalence models presented here were derived from the culture results of each calf within the pen, the significant results observed establish a baseline for exploring sampling a subset of calves from a pen for commercial feedlots. Pen-level sampling is introduced as a potential strategy to forecast the likelihood of antimicrobial resistant bacteria at the time of BRD based on the pen-level C/S attributes identified earlier in the feeding period. The AUC values for the models of pen-level prevalence of AMR and resistance at time of treatment were notably better for samples collected at 13DOF than those from 1DOF.

The present study observed calves with macrolide resistant bacteria at time of first BRD treatment were more likely to have been from cohorts that received tulathromycin metaphylaxis than oxytetracycline metaphylaxis, and calves with tetracycline resistant bacteria isolated at time of treatment were more likely to have received oxytetracycline metaphylaxis. These findings are consistent with metaphylaxis increasing selective pressure for AMR (40). Several other studies in feedlot and stocker cattle have also observed the use of macrolide metaphylaxis associated with increases in macrolide resistance at later sampling times (20, 25, 31, 35, 36). In contrast to the present study which observed increased resistance to macrolides and tetracycline, Nobrega et al. (32) reported metaphylaxis with a macrolide at processing resulted in higher MICs to this drug class at reprocessing, whereas use of tetracyclines did not. As macrolides and tetracyclines are two of the most common antimicrobials used for the control and treatment of BRD, maintaining and monitoring their efficacy is essential (41, 42).

The overall effect of metaphylaxis on long-term AMU and AMR is currently unclear and likely dependent on cattle risk categorization (43, 44). Metaphylaxis is an important tool in BRD management. Benefits include decreases in morbidity and mortality, increases in average daily gain (45), reductions of BRD incidence (41), and evidence suggesting reductions in greenhouse gas emissions (44). Another consideration is the personnel time needed and stress placed on cattle if large numbers of animals require re-handling for treatment (46). Therefore, a more comprehensive investigation of metaphylaxis on AMR is needed, including any potential differences between antimicrobial classes used and overall impacts on long-term antimicrobial usage and AMR.

While AST has been recommended to support antimicrobial stewardship in BRD treatment (47), the utility of this information for predicting clinical outcomes for BRD remains limited. Sarchet et al. (16) enrolled 1,026 heifers at high risk of BRD and collected DNP samples at two time points: day 0 following feedlot arrival and prior to metaphylaxis treatment with tulathromycin, and again at time of first BRD treatment. Tulathromycin susceptibility testing of *M. haemolytica* and *P. multocida* isolates collected at day 0 or at first BRD treatment poorly predicted BRD clinical outcomes, suggesting the unreliability of tulathromycin susceptibility testing at these time points to evaluate tulathromycin efficacy in treated calves (16). Together, these studies suggest the need for more comprehensive investigations into the complexity of AMR testing as related to BRD outcomes.

However, while several factors beyond phenotypic susceptibility, such as host immune function, pharmacokinetics and pharmacodynamics, and timing of drug administration (48) likely contribute to BRD treatment failure, the use of an antimicrobial for which laboratory evidence of resistance exists could be injudicious use. Sampling calves during the early feeding period to identify prevalence information that predicts future AMR patters could provide the treatment guidance addressing antimicrobial stewardship goals set forth by the World Health Organization (49).

Similar to previous work, *M. haemolytica* was the most common bacterial pathogen recovered from cattle at time of BRD treatment (15, 29, 50). For calves receiving first treatment for BRD, the most frequently recovered pathogen at 1DOF or 13DOF varied between *M. haemolytica* and *P. multocida* depending on year and injectable metaphylaxis administered. This could be due to differences in efficacy of tulathromycin and oxytetracycline against these bacteria in this population of animals. *H. somni* was repeatedly recovered more often at time of BRD treatment than at 1DOF or 13DOF. However, this is also consistent with the natural epidemiology of *H. somni* being more prevalent in the post-arrival period (21, 31, 51, 52). Moreover, as part of the broader investigation wherein all cattle within the population were sampled, there was a significant increase in *H. somni* recovery over time from 1DOF to 36DOF across all years with an averaged prevalence of 58% of calves with *H. somni* at 36DOF (21). In comparison, *H. somni* was recovered from 28% of calves at first treatment for BRD.

There were several limitations to the present study. The relatively large total number of exploratory analyses create a risk of false positive results. To protect against this, for each outcome and time frame, only a very specific list of C/S results were considered. The results were then summarized into tables providing an overview of all findings for individual and group level data to allow for detection of trends as well as highlighting more spurious results. While these numbers of models do present a risk, they also reflect the richness of the data generated by this study in terms of C/S outcomes, the collection and laboratory analysis of samples from all calves at relevant time periods, the collection of samples at 1DOF, 13DOF and BRD treatment, and the choice to examine whether C/S data collected early in the feeding period might predict C/S data at BRD treatment.

The relatively low number of BRD morbidities limited the power to detect associations with C/S results. Additionally, time from sample collection to laboratory processing was typically slightly higher for BRD samples compared to those collected at 1DOF, 13DOF, and 36DOF, possibly limiting recovery rates and affecting comparability of bacterial recovery rates at standard time points with those of BRD samples. While a pen size of 100 cattle and a total of 1,599 animals were used in this research, commercial feedlots typically contain 200–300 cattle per pen; this larger number could impact the expected risk of transmission within pens.

The prevalence of AMR bacteria was also low although comparable to on arrival reports for similar cattle and placement times (40). This limited the power of potential predictors at 1DOF. Additionally, the differences in AMR following the two metaphylaxis options resulted in few or no observations of resistance for pen cohorts that were administered the alternate antibiotic classes. For example, in examining risk of macrolide resistance in BRD-treated calves given their status at 13DOF, the finding that no calves had macrolide resistance at 13DOF from 2021 oxytetracycline-treated groups resulted in the failure of the GEE models including design variables to converge. In the cases noted in the results, GEE without adjustment for metaphylaxis or exact logistic regression was used. However, given the low frequency of outcomes of interest in these specific analyses, the potential extent of bias introduced by either not accounting for clustering or by reporting unadjusted value in these few instances was limited.

Calves were presumed to be healthy on arrival, and rectal temperatures were not taken at arrival. Consequently, some calves might have already developed BRD by the time of the first sampling, potentially influencing the predictability of the outcomes of interest. However, this study aimed to replicate standard commercial feedlot practices for high-risk animals receiving metaphylaxis. In such scenarios, commercial feedlots typically do not conduct temperature checks on all arriving calves while concurrently administering metaphylaxis treatments.

Identification of BRD-affected cattle based on clinical signs alone has limited sensitivity due to the inherent nature of cattle to hide evidence of illness. Further, the ability of pen-checkers to accurately identify BRD-affected cattle based on clinical signs had an estimated sensitivity of 0.27 (95% Bayesian credible interval: 0.12–0.65) and specificity of 0.92 (0.72–0.98) (53). Therefore, some truly sick cattle in this study might have been undetected. This study followed cattle for the first 45DOF. While a high proportion of BRD occurs during this time in higher risk cattle (38), results of this study might not be applicable to BRD later in the feeding period.

Lastly, cattle remained in their pen cohorts for the entirety of the study. The generalizability of results observed from this study might not be applicable to pens where re-assortment of cattle has occurred or BRD-treated calves were relocated to hospital pens.

BRD is described as a polymicrobial disease (54). Additionally, as BRD is theorized to be instigated by a viral infection followed by secondary bacterial infections, the recovery of both viruses and bacteria on arrival may be more successful at forecasting associations with future BRD than monocultures. Therefore, studies continuing to explore the impact of co-infections on future BRD risk are warranted.

Another area deserving of future research is the timing of sample collection in relation to the antimicrobial used for metaphylaxis. This study used a second sampling time around two weeks on feed, which aligned well the maximum PMI for tulathromycin. However, the recommended PMI for other long-acting antimicrobials may influence the timing of when nasal microflora stabilize and would be likely to have been shared among pen mates. Optimal sampling times would need to be determined as they pertain to other antimicrobials used for metaphylaxis, as well as in the event of no metaphylaxis. Newer diagnostic tools advertising shorter turnaround times to results than traditional C/S (55) could also improve the feasibility of using laboratory analysis to inform management practices.

Lastly, it would be prudent to explore both the benefits and ramifications associated with animal sampling within the context of cattle health and the financial considerations within a commercial feedlot setting. While both pen and individual calf C/S results were associated with many subsequent outcomes of interest, sampling all calves within a pen to use either the individual or pen-level data would not be practical as a routine recommendation. One potentially more reasonable approach within a commercial setting would entail sampling a smaller, representative subset of the pen. The information gained from sampling 20 to 30 calves from a pen of 200 was explored and discussed in Abi Younes et al. (21). In the instance examined in this study, where tulathromycin is used on arrival and the median days of feed for treatment was 24, samples collected from a random sample of calves on day 13, or perhaps a few days earlier, could provide valuable data to inform antimicrobial decisions for BRD therapy from even a subset of feedlot pens.

Together, the present study, alongside findings from Abi Younes et al. (21), provide foundational understanding of not only sampling a subset of cattle to estimate pen-level prevalence of nasopharyngeal bacteria and antimicrobial resistance status of a feedlot pen, but also the potential to use this pen-level data to estimate the probability of future disease and the resistance profiles of bacteria of interest. However, any sampling effort requires personnel, time and a financial commitment. In addition, re-handling cattle within two weeks on feed, regardless of the number of animals sampled, will inherently stress calves during a period when they are acclimating to the feedlot environment and are susceptible to BRD. Therefore, while the sampling near two-weeks on feed offered insight into BRD risk and antimicrobial susceptibility status at time of BRD treatment, additional research is necessary to examine to what extent that information might improve treatment outcomes or antimicrobial stewardship efforts.

## 5 Conclusion

This research highlights the potential of pen-level C/S results obtained during the early feeding period (1DOF and 13DOF) to predict future likelihood of BRD and C/S results at the time of BRD treatment. Most notably, a higher pen-level prevalence of macrolide or tetracycline resistance, at either 1DOF or 13DOF, was associated with the recurrent recovery of bacteria exhibiting these antimicrobial resistance patterns from calves at the time of first BRD treatment. Laboratory data from strategic pen-level sampling could be available before the peak of expected BRD cases, particularly following metaphylaxis with tulathromycin or other long-acting products, and inform antimicrobial use protocols.

## Data availability statement

The raw data supporting the conclusions of this article will be made available by the authors, without undue reservation.

## Ethics statement

The animal study was approved by University of Saskatchewan Animal Care Committee (AUP 20190069). The study was conducted in accordance with the local legislation and institutional requirements.

## Author contributions

JA: Data curation, Formal analysis, Project administration, Writing – original draft, Writing – review & editing. JC: Conceptualization, Funding acquisition, Writing – review & editing. SG: Conceptualization, Writing – review & editing. AW: Writing – review & editing. CW: Conceptualization, Data curation, Formal analysis, Funding acquisition, Investigation, Methodology, Project administration, Resources, Supervision, Validation, Writing – original draft, Writing – review & editing.
