# Supplementary material for: Association between respiratory disease pathogens in calves near feedlot arrival with treatment for bovine respiratory disease and subsequent antimicrobial resistance status
Source: Front Vet Sci. 2024 Jul 23;11:1416436. doi: 10.3389/fvets.2024.1416436 (PMC11300316; doi:10.3389/fvets.2024.1416436)
Supplement: Supplementary file 2 [file Data_Sheet_1.pdf]

Supplementary Material

Association between respiratory disease pathogens in calves near feedlot arrival with treatment for bovine respiratory disease and subsequent antimicrobial resistance status

Jennifer N. Abi Younes[1], John R. Campbell[1], Sheryl P. Gow[2], Amelia R. Woolums[3], Cheryl L. Waldner[1*]

[1]Department of Large Animal Clinical Sciences, Western College of Veterinary Medicine, University of Saskatchewan, Saskatoon, SK, Canada

[2]Canadian Integrated Program for Antimicrobial Resistance Surveillance, Public Health Agency of Canada, Saskatoon, SK, Canada

[3]Department of Pathobiology and Population Medicine, College of Veterinary Medicine, Mississippi State University, Mississippi State, MS, United States

*** Correspondence:**
Cheryl L. Waldner
cheryl.walder@usask.ca

1 Supplementary Tables

Table S0. Bovine respiratory disease clinical scoring system used to identify calves eligible for first treatment. Adapted from Step *et al.* (24).

BRD Clinical Scoring System – Reference List			
**Score**	**BRD Code**	**Key Clinical Signs** (*one or more signs are observed)	**Occasional Clinical Signs** (*may also be observed)
**0**	**Normal**	▪ Clinically normal animal	
**1**	**Mild BRD**	▪ Mildly ↑ respiratory rate<br>▪ Mild – moderate gauntness<br>▪ Quiet when observed from afar, but **becomes alert when sees a human**<br>▪ Shallow or dry cough	▪ Nasal discharge (clear, cloudy, white, or yellow)

BRD Clinical Scoring System – Reference List			
**Score**	**BRD Code**	**Key Clinical Signs**<br>(*one or more signs are observed)	**Occasional Clinical Signs**<br>(*may also be observed)
**2**	**Moderate BRD**	▪ Mild or moderate depression<br>· **lethargic**, but may become alert when approached<br>· lower than normal head carriage<br>· “hiding” behavior<br>▪ **Mild – moderate muscle weakness**<br>· droopy ears<br>· slow stepping or mild incoordination<br>▪ **Coughing repeatedly**<br>▪ Breathing with **slightly ↑ abdominal effort**<br>▪ Moderate gauntness	▪ ↑ Respiratory rate<br>▪ Nasal discharge (clear, cloudy, white, or yellow)
**3**	**Severe BRD**	▪ Severe depression or weakness<br>· **when approached: does not become alert**, low head carriage maintained, does not move away<br>· cross stepping<br>▪ Repeated **deep cough**<br>▪ **Marked respiratory effort**<br>· open mouth breathing or panting<br>· moderate – marked ↑ abdominal effort	▪ ↑ Respiratory rate<br>▪ Nasal discharge (clear, cloudy, white, or yellow)<br>▪ Moderate – extreme gauntness
**4**	**Moribund** (near death)	▪ **Down, does not rise** when approached or stimulated<br>▪ Standing, but **does not move unless directly stimulated**<br>· very weak: drags feet, sways, stumbles, falls<br>▪ **Eyes very sunken, abdomen very tucked up**	▪ Any signs described for scores 1, 2, or 3

BRD Case Definition	
**BRD Score**	**Criteria**
1 – 2	▪ AND a rectal temperature ≥ 40 °C
≥ 3	▪ Regardless of rectal temperature<br>▪ With NO other obvious signs of disease (e.g., lameness, diarrhea, swollen limbs, etc.)

Table S1. Overview of statistical analyses for calves sampled near 36DOF for all culture and susceptibility (C/S) test results considered associated with C/S results for samples collected at 1DOF and 13DOF for individual calf-level, pen-level, and contextual analysis.

	**Individual outcomes & risk factors**	**Pen-level outcomes & risk factors**
**Risk period for outcome of interest:**	**Outcome DNP C/S - Calf status 36DOF**	**Outcome DNP C/S - Pen results 36DOF**
**36DOF**	Individual calf results from C/S at 1DOF	Pen results from calf C/S at 1DOF
**36DOF**	Individual calf results from C/S at 13DOF	Pen results from calf C/S at 13DOF

Culture and susceptibility (C/S) results considered:

- recovery of *M. haemolytica*, *P. multocida,* or *H. somni*
- co-isolation of two or more bacteria
- recovery of any BRD bacteria with: any AMR, macrolide resistance, or tetracycline resistance

Table S2. For calves sampled near 36DOF, associations between culture and susceptibility (C/S) results for calves at 1DOF and likelihood of subsequent C/S results near 36DOF. *n*=309 calves (tag identification not available for one calf at 1DOF). Analysis adjusted for year and injectable metaphylaxis administered and accounted for clustering by pen cohort.

			95% CI		
**Risk Factor: Culture Positive at 1DOF**	**Outcome: C/S result near 36DOF**	**OR**	**Lower**	**Upper**	***P*-value**
M. haemolytica	*M. haemolytica*	1.0	0.6	1.8	0.96
P. multocida	***P. multocida***	**2.0**	1.1	3.7	**0.02**
H. somni	*H. somni*	0.6	0.2	1.4	0.23
Any bacteria with AMR	Any bacteria with AMR	1.2	0.3	4.3	0.83
Any bacteria with macrolide resistance*	Any bacteria with macrolide resistance		Not estimable		
**Any bacteria with tetracycline resistance****	**Any bacteria with tetracycline resistance**	**16**	2.4	∞	**0.008**

*Zero calves sampled near 36DOF had bacteria with macrolide resistance recovered at 1DOF.

**Exact logistic regression equation (SAS® version 9.4, Cary, NC, USA).

Table S3. For calves sampled near 36DOF, associations between culture and susceptibility (C/S) for calves at 13DOF and likelihood of subsequent C/S at 36DOF. Analysis adjusted for year and injectable metaphylaxis administered and accounted for clustering by pen cohort. AST data were not available for one calf at time point 3 (pen 16); *n*=309 calves for AMR analyses (N=16 pens).

			**95% CI**		
**Risk Factor: Culture Positive at 13DOF**	**Outcome: C/S result near 36DOF**	**OR**	**Lower**	**Upper**	***P*-value**
M. haemolytica	*M. haemolytica*	1.2	0.7	1.9	0.48
P. multocida	***P. multocida***	**6.8**	**3.1**	**15**	**<0.001**
H. somni	*H. somni*	1.0	0.3	3.4	0.97
Any bacteria with AMR	Any bacteria with AMR	1.9	0.8	4.2	0.13
Any bacteria with macrolide resistance*	Any bacteria with macrolide resistance	1.2	0.5	2.7	0.62
Any bacteria with tetracycline resistance	Any bacteria with tetracycline resistance	3.1	0.9	11	0.08

*Unconditional GEE reported as zero calves sampled near 36DOF from year 2021/oxytetracycline cohorts had bacteria with macrolide resistance recovered.

Table S4. For calves sampled near 36DOF, associations between culture and susceptibility (C/S) results at 36DOF given pen-level prevalence of C/S results at 1DOF and 13DOF. Analysis adjusted for year and metaphylaxis and accounted for clustering by pen cohort. *n*=1,599 calves at 1DOF and 1,596 calves at 13DOF (N=16 pens) (SAS® version 9.4, Cary, NC, USA).

Risk Factor: Pen-level C/S Prevalence	Outcome Pen Probability	OR	95% CI Lower	95% CI Upper	*P*-value
**Increase in prevalence at 1DOF**	**C/S at 36DOF**				
M. haemolytica (5%)	*M. haemolytica*	1.0	0.9	1.2	0.53
P. multocida (5%)	*P. multocida*	1.0	0.9	1.0	0.39
H. somni (5%)	*H. somni*	1.0	0.9	1.1	0.91
**Tetracycline resistance (5%)**	**Tetracycline resistance**	**1.5**	**1.2**	**1.9**	**0.0007**
Macrolide resistance (5%)*	Macrolide resistance	8.8	0.8	103	0.08
Any AMR (5%)	Any AMR	1.0	0.6	1.8	0.92
**Increase in prevalence at 13DOF**	**C/S at 36DOF**				
M. haemolytica (5%)	*M. haemolytica*	1.0	0.9	1.0	0.23
P. multocida (5%)	***P. multocida***	**1.2**	**1.2**	**1.3**	**<0.0001**
H. somni (5%)	*H. somni*	1.0	0.8	1.3	0.93
**Tetracycline resistance** (5%)	**Tetracycline resistance**	**1.0**	**1.0**	**1.1**	**0.04**
**Macrolide resistance** (5%)*	**Macrolide resistance**	**1.2**	**1.1**	**1.3**	**<0.0001**
**Any AMR** (5%)	**Any AMR**	**1.0**	**1.0**	**1.1**	**0.01**

*Unconditional GEE reported as zero calves sampled near 36DOF from year 2021/oxytetracycline cohorts had bacteria with macrolide resistance recovered.

Table S5. Summary of associations between culture and susceptibility (C/S) results at 1DOF and 13DOF and subsequent C/S for calves at 36DOF.

**Individual calf level models**		
**Predictor**	**Outcome**	**Significant risk factors**
C/S result at 1DOF	C/S result at 36DOF	*P. multocida*, tetracycline resistance
C/S result at 13DOF	C/S result at 36DOF	*P. multocida*
**Pen-level data**		
**Risk factor: Pen-level C/S prevalence**	**Outcome**	**Significant risk factors**
Pen prevalence at 1DOF	C/S result at 36DOF	Tetracycline
Pen prevalence at 13DOF	C/S result at 36DOF	*P. multocida,* any AMR, macrolide resistance, or tetracycline resistance
